# Khat chewing and associated factors among public secondary school students in Harar town, Eastern Ethiopia: a multicenter cross-sectional study

**DOI:** 10.3389/fpsyt.2023.1198851

**Published:** 2023-08-29

**Authors:** Kabtamu Nigussie, Abraham Negash, Addisu Sertsu, Abiy Mulugeta, Aklilu Tamire, Obsan Kassa, Tilahun Abdeta, Jerman Dereje

**Affiliations:** ^1^Department of Psychiatry, School of Nursing and Midwifery, College of Health and Medical Science, Haramaya University, Harar, Ethiopia; ^2^Department of Midwifery, School of Nursing and Midwifery, College of Health and Medical Science, Haramaya University, Harar, Ethiopia; ^3^Department of Nursing, School of Nursing and Midwifery, College of Health and Medical Science, Haramaya University, Harar, Ethiopia; ^4^School of Public Health, College of Health and Medical Science, Haramaya University, Harar, Ethiopia

**Keywords:** prevalence, khat chewing, students, Harar, Ethiopia

## Abstract

**Background:**

Khat is a huge, evergreen tree that grows at high altitudes throughout the Arabian Peninsula and in the region stretching from eastern to southern Africa. Cathinone, cathine, and norephedrine are psychoactive ingredients contained in khat. Ethiopian teenagers, especially those in secondary school, frequently use khat. This use of khat may lead to students frequently missing class and experiencing subpar academic performance. However, the study area lacks information regarding the prevalence of khat use and the factors associated with it.

**Objective:**

This study's primary goal is to determine the prevalence of khat chewing and related factors among secondary school students in public schools in Harar, Eastern Ethiopia, in 2022.

**Methods:**

A multicenter cross-sectional study design was employed from June 01–June 30, 2022, in three public secondary schools in Harar town in a sample of 485 students. Systematic random sampling was used to choose the study sample. Data were gathered using self-administered questionnaires, and the Alcohol, Smoking, and Substance Involvement Screening Test (ASSIST) was used to assess khat chewing. Epidata version 4.6 was used to enter the data, while STATA version 14 was used to analyze them. To determine the factors related to khat chewing, bivariate and multivariate logistic regression analysis was conducted, and statistical significance was determined at a 95% confidence level with a *P*-value under 0.05.

**Results:**

Out of 485 eligible participants, 455 responded to this survey, giving a response rate of 93.8%. Overall, 33.2% (95% CI: 29.2%−37.6%) of the sample's participants reported currently chewing khat. Age ranged from 20 to 25 years (AOR = 2.04; 95% CI: 1.19–3.48), male students (AOR = 7.03; 95% CI: 4.35–12.57), current alcohol user (AOR 6.48; 95% CI: 2.30–18.28), presence of chewer friends (AOR 3.86; 95% CI: 2.38–6.24), and depression (AOR 1.84, 95%CI: 1.02–3.30), were strongly associated with khat chewing at a *p*-value of < 0.05.

**Conclusion:**

Khat chewing was very common among students in Eastern Ethiopia's public secondary schools. Ages between 20 and 25 years, being a male, being current alcohol users, having chewer buddies, and depression are all significantly linked to khat use. Thus, schools should create and implement audience-specific behavioral change communication to deter and stop students from chewing khat. Additionally, it is important to ban the sale of khat to young adults and adolescents, promote medical care for khat users, and foster peer advocacy for support services.

## Introduction

Catha edulis (khat) is a stimulant drug that is cultivated in Yemen and most of East Africa (1). It is a plant or tree with leaves imported into other countries but has long been chewed in eastern Africa and the Arabian Peninsula (2). Chewing khat is prohibited in some nations, such as the US, and is considered a controlled substance in Canada (3), but it is legal in some European countries (4).

Students chew khat because it stimulates their brains due to the presence of stimulatory ingredients in the herb's fresh leaves, such as cathinone, cathine, and norephedrine (5). Cathinone is a powerful stimulant (6) that stimulates the central nervous system, similar to how amphetamine does (7). Cathine is a milder form of cathinone (8, 9). Students, particularly those in higher education and those studying for extended periods, chew khat leaves because they act as stimulants (10).

Khat chewing is common in Africa, mostly in countries in the Horn of Africa (11–13). Khat consumption has a negative impact on family and social life (14, 15). It may act as a factor that exacerbates family disruption (16). Khat is a legal drug like tobacco, cigarettes, and alcohol in Ethiopia, openly sold at markets and chewed on the streets.

There is currently a global concern over substance abuse and related issues since they might result in mental health disorders, which account for 14% of the global illness burden (17). In the world, a few million people chew khat; 10 million people are thought to do so. A cross-sectional survey conducted in the Jazan region, southwest of the Kingdom of Saudi Arabia (KSA), revealed that the prevalence of khat chewing was 21.4%. Khat chewing prevalence was high in secondary schools (21.5%) compared to colleges (15.2%) (18). With the arrival of immigrants from Africa and the Middle East, khat chewing has spread to a number of Asian and European nations, as well as Australia and the United States (19). In the study conducted in Kenya, the current prevalence of khat chewing was 36.8%, with a male gender predominance (54.8%) (20). In Ethiopia, 15.3% of people aged 15 to 49 used khat, with men using it at a rate of 22.6% and women at a rate of 9.1% (21).

A 2011 report from the Ethiopian Demographic and Health Survey (EDHS) showed khat chewing was more common in the Eastern, Central, and Northeastern parts of the country; the highest wealth index quintile, older age group, unskilled workforce, rural residents, exposure to mass media, and administrative regions were factors statistically associated with khat chewing practice (22).

A high prevalence of khat chewing has been reported in different parts of Ethiopia, from 6.95% to 64.9% in Oromia and Amhara regions, respectively (10), which was mainly related to spiritual (23) and cultural practices (24). Increasing intake of psychoactive substances like khat among young people as part of daily habits has also been reported (25). The beliefs that khat may enhance concentration (26), performance motivation, and socialization attract many adolescents and secondary school students to consume khat (27).

The lifetime prevalence of khat use among secondary students of Ethiopia was indicated at 15.4% in Northern Shewa Ethiopia, Northwest Ethiopia (19.6%), Southeast Ethiopia (23.6%), and Eastern Ethiopia (24.2%) (28–31). According to the study findings, a remarkable proportion of students used khat where it was more available. A community-based study in Southwest Ethiopia showed that youths were more accustomed to chewing khat than other groups of populations (32).

According to reports, the consumption of khat can have negative effects on both physical and mental health (33). It can be a risk factor for various issues such as elevated blood pressure, rapid heartbeat, sleep disturbance, anorexia, gastrointestinal symptoms like constipation, inability to void, restlessness, impaired sexual dysfunction, which is potency in men, and hallucination (34). Moreover, it causes excessive chattiness, a heightened sense of energy, alertness, improved attention, a faster heartbeat and breathing rate, a rise in body temperature and blood pressure, and a decrease in hunger (35). While some research indicates that khat users are more susceptible to khat-related issues like depression, posttraumatic stress disorders (PTSD), and common mental disorders (36), other studies have suggested that khat has traditional medicinal uses for diabetes, depression, muscle strength, a reduction in excessive food intake and sleep deprivation, and an increase in aggression (37).

A study revealed that people who chew khat are less likely to excel academically (38). Although the clear mechanism linking khat chewing to academic performance is unknown, several studies support the idea that khat-chewing students are more likely to perform poorly in their academics or, put another way, are at a higher risk of failing. This underperformance may be attributed to factors such as excessive time spent in khat-chewing sessions, sleep disturbances (insomnia), absenteeism from school, and impaired functioning in the morning following a khat-chewing session (39, 40). Khat consumption can also affect sleep; it can cause late awakening, decreased quality of sleep (41), and be a risk factor for social isolation, family breakdown, and neglect of social responsibilities (42). Eastern Ethiopia is a major producer and exporter of khat in East Africa, and the majority of the country's khat comes from this region (43). Despite this, not enough studies have been conducted to investigate this region's khat chewing customs. Consequently, the primary goal of this study is to determine the prevalence and contributing factors of khat use among students attending public secondary schools in Harar, Eastern Ethiopia.

## Methods and materials

### Study area and period

The study was conducted in Harari regional state from 1 June to 30 June 2022. Harar is the capital city of the Harari regional state in eastern Ethiopia. The city also serves as the East Hararghe Zone's administrative center in the Oromia Region and is located at an altitude of 1,885 m (6,184 ft.), around 526 km from Ethiopia's capital, Addis Ababa. The population (2021 projection based on the 2007 Census, CSA) of the region was estimated to be 270,000, of which 136,000 were men and 134,000 were women. There are 15 schools in Harar town, Harari regional state, Eastern Ethiopia, with a total of 1,1686 students, of whom 5,977 were male and 5,709 were female students. There are seven public secondary schools and eight private secondary schools.

### Study design and population

A multicenter cross-sectional study was carried out. The source population consisted of every secondary school student in Harar town. Students who attended secondary schools in Abidar, Abubaker, and Shek-Abdullah at the time of the study were eligible for inclusion in the study. All students over the age of 15 who could obtain their parent's signed informed consent form were included in the study, and those who were unable to communicate or were very ill at the time the data were gathered were excluded from it.

### Sample size determination

A single population proportion was used to compute the sample size. In this study, we used the 24.2% rate of khat chewing from a survey done in public secondary schools in Eastern Ethiopia (31). Therefore, we have the following equation:


$$\begin{array}{l} \text{n}=\frac{\text{Z}\frac{{\text{α}}^{2}}{2}\text{p}\left(1-\text{p}\right)}{\text{d}2}, \end{array}$$

while n = minimum sample size for the study

Z = the reliability of the coefficient corresponding to 95% (z = 1.96).

p = 24.2% in Eastern Ethiopia. This prevalence was taken from a study conducted at Harar public secondary schools (31).

D = the margin error d = 4%


$$\begin{array}{l} \text{n}=\frac{{\left(1.96\right)}^{2}*\;0.242\left(1-0.242\right)}{{\left(0.04\right)}^{2}}=441, \end{array}$$

As a result, adding a 10% non-response rate resulted in a final sample size calculation of 485.

### Sampling techniques

**Figure 1:**
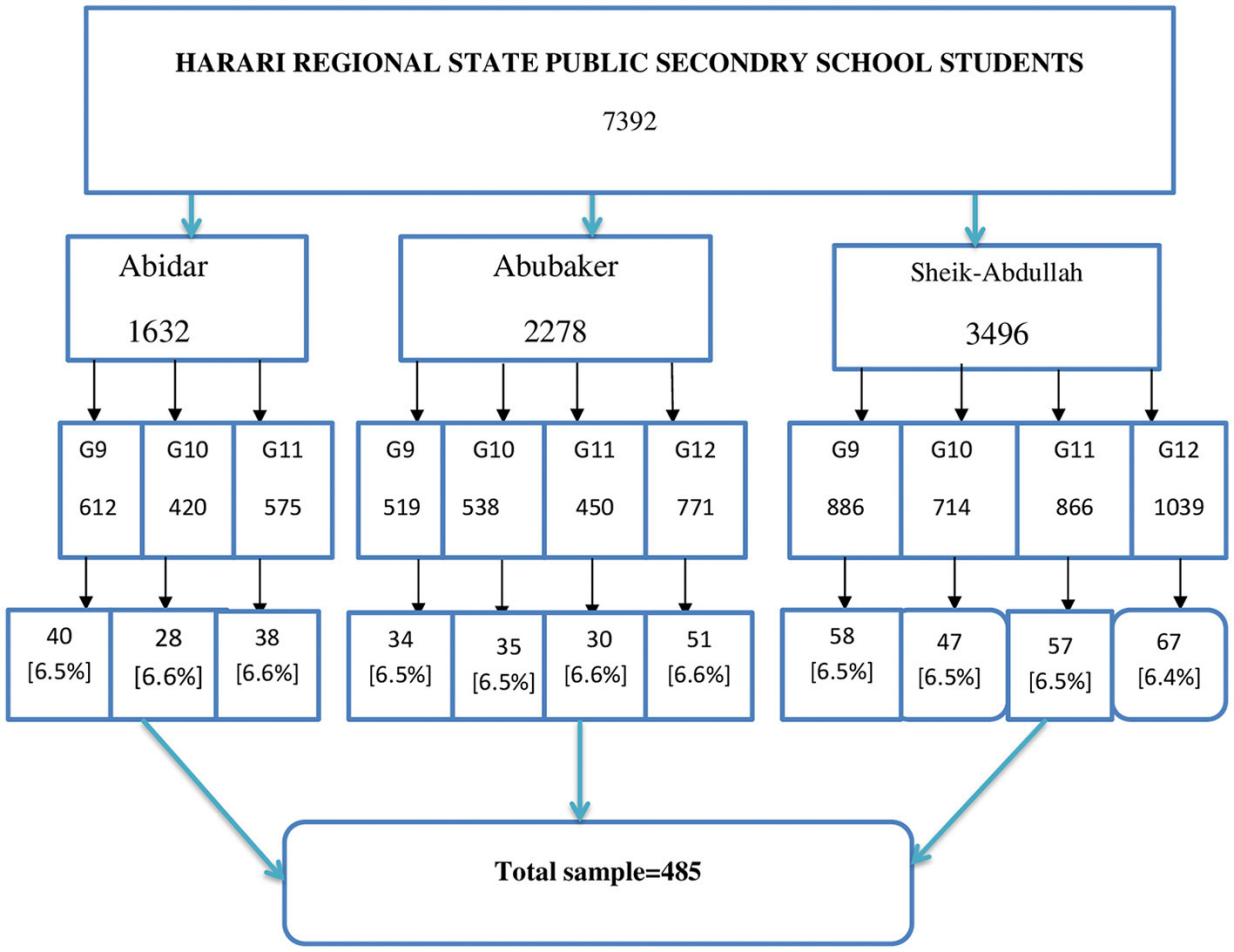
Schematic presentation of sampling techniques to assess prevalence of Khat chewing and associated factors among public secondary schools students, in Harar town, Eastern Ethiopia 2022.

Three secondary schools were selected using simple random sampling, and then, our study populations were stratified according to their school and grade. From each stratum, samples were drawn proportionally by systematic random sampling with an interval of 7,392/485 = 15, K = 15. We took samples from each school proportionally. Abidar Secondary School has a total of 1,632 students for a total sample size of 106. Abubaker Secondary School has a total of 2,278 students, with a sample size of 150 Shek-Abdullah Secondary School has a total of 3,496 students in a sample of 229, so we collected data from our sample according to this proportion from each school, as shown in Figure 1.

### Data collection instruments and procedures

Amharic and Afan Oromo language versions were used to collect data using a structured paper and pen questionnaire. The study's questionnaires consisted of four sections: Sociodemographic information was collected by assessing them using questions developed from the literature. The Alcohol, Smoking, and Substance Involvement Screening Tool (ASSIST) shorter form was used. From the ASSIST tool's eight questions, we only used the first two questions of the tool for three substances: Alcohol, cigarettes, and Khat. We used question one to identify every use and a modified version of question 2, where we modified the responses from never, once, twice, monthly, weekly, or daily to “yes” or “no.” An international team of substance abuse researchers created the Alcohol, Smoking, and Substance Involvement Screening Test (ASSIST) for the World Health Organization (WHO) to identify psychoactive substance use and associated issues in clients receiving primary care (44).

The Oslo social support scale-3 was used to assess the students' social support. The Oslo-3 is a concise and effective tool for assessing the degree of social support. The Oslo-3 solely consists of three questions that inquire about the number of close friends, the perception of other people's concerns, and the relationship with neighbors, with an emphasis on the availability of practical assistance. According to the three-item Oslo social support scale, those who scored 12–14, 9–11, and 3–8 had strong, moderate, and poor social support, respectively (45).

Depression was assessed using the Patient Health Questionnaire (**PHQ-9)**. The PHQ-9 score spans the range of 0 to 27. Scores ranged from 0 (not at all) to 3 for each of the nine items (nearly every day). PHQ-9 scores range from 0 to 27; a score of 0 to 4 indicates minimal or no depression, a score of 5 to 9 shows mild depression, a score of 10 to 14 suggests moderate depression, a score of 15 to 19 indicates moderately severe depression and a score of 20 to 27 indicates severe depression (46). Furthermore, PHQ-9 demonstrated a specificity and a sensitivity of 67% and 86%, respectively, in the setting of Ethiopian healthcare. For depression screening, a cut-off value of 10 or higher has been employed (47).

Generalized Anxiety Disorder Seven-Item (GAD-7) was utilized to assess anxiety symptoms. The response options of “not at all,” “a few days,” “more than half the days,” and “almost every day” were given scores of 0, 1, 2, and 3, respectively, and the scores for the seven questions were totaled up. The GAD-7′s sensitivity and specificity for GAD are 89% and 82%, respectively, when using a threshold score of 10 (48).

### Study variables

#### Outcome variable

Regular Khat chewing (Yes/No).

#### Independent Variables

Sociodemographic factors (such as gender, age in years, marital status, religion, living situation, grade level, and monthly income in Ethiopian Birr), clinical factors (such as depression and anxiety), other substance-related factors (such as current and lifetime alcohol, tobacco, and other substance use), and psychosocial factors (such as perceived social support) were all taken into consideration.

#### Operational definitions

**Current substance use**: According to the Alcohol, Smoking, and Substance Involvement Screening Tool (ASSIST), at least one of the following substances (alcohol, tobacco, or cigarettes) was used for non-medical purposes within the previous 3 months (49).

**Ever substance use**: We used one or more specified drugs (such as alcohol, khat, or cigarettes) for non-medical purposes at least once throughout one's lifespan, as per ASSIST (50).

**Social support:** Individuals who scored 12–14, 9–11, and 3–8 had strong, moderate, and poor social support, respectively, using the 3-item Oslo social support scale (45).

**Regular Khat chewing:** For the previous year or longer, khat was chewed at least once every week (51).

**Lifetime Khat chewer:** An individual who has ever used khat at least once in their lifetime (52).

**Cigarette smoker:** This term was used to describe those who smoked cigarettes at least once a week in the previous month (30 days) (53).

**Current alcohol user:** This term was used to define drinking alcohol for a reason other than medical in the past three months (54).

**Depression**: Those with a PHQ-9 score of 10 or higher were determined to have depression. (55).

**Anxiety**: Those with a GAD-7 score of 10 or higher were determined to have anxiety (48).

### Data quality control

Two language experts translated the original English version of the survey into an Afan Oromo and Amharic language version that was suitable for the participants. After forward and backward translation, we conducted a pilot test among 23 respondents, as initial validation and internal consistency were also checked by Cronbach's alpha. This study pretest was conducted done to check the suitability of tools such as ASSIST, PHQ-9, and GAD-7, and their Cronbach alpha values were 0.81, 0.79, and 0.84, respectively. Prior to data collection, the lead investigators trained data collectors and supervisors on how to use questionnaires, the ethical value of confidentiality, and data administration. Daily supervision was provided to the data collectors, and the lead investigators and supervisors reviewed the completed questionnaires to ensure they were accurate each day.

### Data processing and analysis

Epi-Data version 4.6 was used to code, check, and enter the data before being exported to STATA version 14 for analysis. Descriptive statistics were used to examine the sociodemographic details and other aspects of the respondents. A bivariable logistic regression analysis was conducted to determine the relationship between each independent variable and the outcome variable. The multivariable logistic regression model included all variables whose *p*-value was < 0.2 in the bivariate logistic regression analysis. The adjusted odds ratio (AOR) with a 95% confidence interval (CI) was calculated when the *p*-value was < 0.05, which was declared statistically significant. Using the Hosmer-Lemshow test, the model's fitness was tested for goodness.

### Ethical consideration

Ethical clearance was obtained from Haramaya University College of Health and Medical Science's Institutional Health Research Ethics Review Committee (IHRERC) reference number (IHRERC/055/2022). All participants received written agreements clarifying the study's goals and their right to decline. Participants in the study were also made aware of their ability to refuse to answer any questions. Without using any bias, every participant was chosen at random. Access to the results was strictly restricted to the group members, and completed surveys were handled with care. To protect the respondents' confidentiality, anonymity was preserved. Before participating in the study, each subject received written consent/assent in Amharic and Afan Oromo and signed it. Since the instruments that we used in our study are public domain measures or free to users, they are downloadable from the website, and no permission was required from copyright holders to reproduce, translate, display, and distribute them.

## Results

### Sociodemographic characteristics of respondents

Among the 485 selected samples, 455 participants were included in the study, with a response rate of 93.8%. The study excluded 30 participants. Around 22 individuals were unable to bring signed informed consent from their parents, and eight participants were unable to communicate owing to a serious illness. Additionally, none of the study participants' responses were missing. The interquartile age range of our respondents was 17–19 years, with a median age of 18 years. Of these, 227 (49.9%) were women, resulting in a sex ratio close to 1:1. A majority of 351 respondents (77.1%) were aged between 15 and 19 years. Half (50%) of the participants were Muslim. Nearly all, 440 (96.7%), were unmarried. Regarding the grade level of our participants, approximately 159(34.9%) of our participants were in grade 11, while 110 (24.2%) were in grade 12 (Table 1).

**Table 1:** Sociodemographic characteristics of the sampled public secondary school students in Harar town, Eastern Ethiopia, 2022 (*n* = 455).

**Variable**	**Frequency**	**Percent**
**Sex**		
Male	228	50.1
Female	227	49.9
**Age**		
15–19	351	77.1
20–25	104	22.9
**Religion**		
Muslim	265	58.2
Orthodox	149	32.7
Other^*^	41	9
**Marital status**		
Unmarried	440	96.7
Married	15	3.3
**Grade**		
Grade 9	91	20
Grade 10	95	20.9
Grade 11	159	34.9
Grade 12	110	24.2
**Residency**		
Urban	391	85.9
Rural	64	14.1
**Living arrangement**		
With family	441	96.9
Alone	14	3.1
**Income**		
1,000–3,000	177	38.9
3,001–5,000	182	40
5,001–7,000	68	14.9
above 7,000	28	6.2

Other^*^: Catholic, Hawariat.

### Clinical, other substance, and social support characteristics of the respondents

Of the total participants, 455 (5.7%) used alcohol in their lifetime, and 23 (5.1%) of our participants reported smoking cigarettes in their lifetime. Out of 455 study participants, 89 (19.6%) and 94 (20.7%) participants reported depression and anxiety, respectively. In terms of social support, 76 participants (16.7%) had strong social support, compared to 195 individuals (42.9%) with moderate social support and 184 participants (40.4%) with poor social support (Table 2).

**Table 2:** Clinical, other substance, and social support characteristics of public secondary school students in Harar town, Eastern Ethiopia 2022 (*n* = 455).

**Variable**	**Frequency**	**Percent**
**Alcohol**		
Yes	26	5.7
No	429	94.3
**Cigarette**		
Yes	23	5.1
No	432	94.9
**Depression**		
Yes	89	19.6
No	366	80.4
**Anxiety**		
Yes	361	79.3
No	94	20.7
**Social support**		
Poor	184	40.4
Moderate	195	42.9
Strong	76	16.7

### Prevalence of khat chewing

The results of this study showed that 163 (35.8%); 95% CI (32.0%−40.6%) people had ever chewed khat, and 151 (33.2%) of them were current khat chewers.

### Factors associated with khat chewing

Age, marital status, religion, living situation, place of residence, level of education, and current cigarette smoking were all revealed to be factors that were related to khat use in bivariate logistic regression analysis. According to the multivariable logistic regression model, there is a substantial correlation between khat chewing and being between the ages of 20 and 25 years, being male, living with khat chewers, being current alcohol drinkers, and experiencing depression. According to this study's findings, chewing khat has no association with social support or anxiety symptoms. As a result, students who consume alcohol have a 6-fold higher chance of chewing khat than those who do not (AOR = 6.48; 95% CI: 2.30–18.28). Compared to female students, male students were seven times more likely to chew khat (AOR = 7.03; 95% CI: 4.35–12.57). The likelihood of chewing khat was four times greater for students who lived with khat users than those who did not (AOR = 3.86; 95% CI: 2.38–6.24). The odds of students chewing khat doubled as students' ages increased (AOR = 2.04; 95% CI: 1.19–3.48). Students who were depressed were twice as likely to use khat as those who were not depressed (AOR = 1.84, 95%CI: 1.02–3.30) (Table 3).

**Table 3:** Bivariate and multivariate factors associated with Khat chewing among public secondary school students in Harar town, Eastern Ethiopia 2022 (*n* = 455).

**Variable**	**Khat chewing**		**COR (95%CI)**	**AOR (95%CI)**
	**Yes**	**No**		
**Age**				
15–19	96	255	1.00	1.00
20–25	55	49	2.98 (1.90, 4.68)	**2.04 (1.19, 3.48)** ^*^
**Sex**				
Male	120	108	7.03 (4.44, 11.12)	**7.39 (4.35, 12.57)** ^**^
Female	31	196	1.00	1.00
**Marital status**				
Single	142	298	1.00	1.00
Married	9	6	3.15 (1.10, 9.02)	0.45 (0.11, 1.77)
**Residency**				
Urban	116	275	1.00	1.00
Rural	35	29	2.86 (1.67, 4.90)	1.60 (0.83, 3.06)
**Presence of chewer friends**				
Yes	98	90	4.40 (2.90, 6.66)	**3.86 (2.35, 6.24)** ^**^
No	53	214	1.00	1.00
**Current alcohol users**				
Yes	19	7	6.12 (2.51, 14.88)	**6.48 (2.30, 18.28)** ^**^
No	132	297	1.00	1.00
**Depression**				
Yes	38	51	1.67 (1.04, 2.68)	**1.84 (1.02, 3.30)** ^*^
No	113	253	1.00	1.00

1.00, Reference, AOR, Adjusted odd ratio.

* *p*-value < 0.05,

** *p*-value < 0.001, Chi square = 5.04; df = 8, and Hosmer-Lemshow test = 0.56.

## Discussion

This study sought to identify the prevalence and risk variables for public secondary school students in Harar town.

The age range between 20 and 25 years, male gender, current alcohol usage, the presence of the chewer's friends, and depression were the independent predictors of current khat use. Notwithstanding the negative repercussions of khat use on one's health, money, and society (56–58), the community in eastern Ethiopia cultivates and uses khat in large quantities (59).

In this cross-sectional survey of khat chewing among public secondary school students in Harar town, the prevalence of current and lifetime khat chewing was 33.2% and 35.8%, respectively. This is higher than the previous study conducted in Sidama zone, southern Ethiopia, at 13%(40), the prevalence of 24.2% found in a study conducted in Eastern Ethiopia (53), Gondar, Northwest Ethiopia, which was 22.7% (60), Kolfe-Keraniyo sub-city, Addis Ababa, which was 9.4% (61), and Jima high school students, which was 14.2% (62). The supply of khat is likely lower in the northern and central regions of Ethiopia than in the eastern region of Ethiopia, which could account for the disparity. Furthermore, the gap might be explained by the differences between study participants (secondary school vs. university students), but this needs more research. Another reason for the gap could be that parents and the school community as a whole, including students, are less aware of the impact of khat on social, psychological, and physical wellbeing. However, more research is required.

In addition to this, there are a number of other causes for the reported variations in current khat chewing, including variations in sample size and cultural variations in perceptions of chewing frequency. The sample size of a study conducted in Sidama zone (40), Jimma town (62), and Addis Ababa (61) was lower compared to the sample size of this study. In certain societies, especially in eastern Ethiopia, khat consumption is accepted as an integral part of their culture, along with birth rituals, circumcision, and marriage events. Similarly, clan elders and religious devotees also frequently consumed khat for a similar reason: all-night sessions of prayer during Ramadan, which have been chewed with their children over a long period of time as cultural norms (63). Khat consumption is part of cultural norms in Eastern communities, which may also involve family interactions, parenting styles, practices, family modeling, and family backgrounds that can influence adolescent and youth behavior (63).

According to the current study, chewing khat is strongly related to the male sex, which is corroborated by Saudi Arabian research (18) among Ataye secondary school students (29), Bale Zone, Oromia Regional State (28), Jimma city (62). This may be a result of men's propensity to take drugs more frequently than women do, as well as the societal acceptance of men using drugs in Ethiopia and other nations that use khat. According to a cross-sectional study conducted among secondary school students in the Sidama zone, Southern Ethiopia, drinking alcohol is also linked to a high incidence of chewing khat (40). Possible explanations might be that there is a higher tendency to abuse more than one substance if someone is addicted to a specific substance.

Khat chewing is also connected to having chewer friends around. This finding is supported by a previous cross-sectional study conducted at a secondary school in the eastern part of Ethiopia (31). This influence of individuals living with them, sharing behavior, and normalizing the habit might contribute to this association. Our study indicates that the age range between 20 and 25 is linked to current khat chewing, which was supported by the cross-sectional study conducted on two secondary schools in Gondar, Northwest Ethiopia (60). This might be due to young people struggling to control their emotions and make logical decisions due to the underdeveloped prefrontal cortex, which can be dangerous when combined with offers to consume drugs. Additionally, most young people can be influenced by many external and internal factors. This emotional upheaval makes adolescents more vulnerable to Khat chewing than their counterparts.

In this study, khat use and depression are correlated. An earlier study undertaken in Bahirdar, Northwest Ethiopia, supports this finding (52, 64). Khat may stimulate adrenocortical function as an explanation. The main psychoactive components of khat, cathinone, and cathine cause the release of cortisol, norepinephrine, and dopamine. As a result, the respondents first exhibit psycho-stimulatory effects, including enthusiasm and chattiness. Then, they experience constant anxiety, depression, and tension (33). Another explanation is socioeconomic issues caused by the increased need for cash to purchase khat. According to this study's findings, khat use has no association with social support or anxiety symptoms.

### Strengths and limitations of the study

The study's greatest strengths are utilizing a representative sample size and a proper sampling method. The study provides useful information that will aid policymakers in creating a strategy to reduce the prevalence of the practice of chewing khat and its detrimental impacts on society and health. Due to the cross-sectional nature of this study's design, a temporal relationship between the outcome and the independent variable cannot be established. Relying on one's own judgment to handle delicate situations might lead to social desirability bias, which understates the use of khat chewing. There is a need for additional research employing a more representative sample of teenagers in the nation because the study was conducted in schools and cannot be generalized to all youth in Ethiopia.

### Clinical implications of the study

Chewing khat makes it more likely that students will frequently be absent from school and have subpar academic performance. By estimating prevalence and predicting factors, this study may make a significant contribution to enhancing mental health services provided to public secondary school students in Eastern Ethiopia who use drugs. This study will further encourage researchers and clinical practitioners to examine the study population for substance use disorders and related concerns.

## Conclusion and recommendation

In eastern Ethiopia, secondary school students' khat chewing is highly prevalent. Ages of 20 to 25 years old, male gender, current alcohol users, the existence of chewers as friends, and depression are all significantly linked to khat use. This research suggests that early intervention, aimed at pre-secondary and secondary school students, is necessary to lessen the negative effects of khat use on one's health, finances, and social life. Adolescent substance use prevention programs should also cover parental substance use. Strategies to decrease khat chewing among secondary school students should be designed to mitigate the further consequences of this substance. The strategies may include developing peer education programs, creating awareness among secondary school students, and implementing measures to keep khat selling houses away from school.

Moreover, preparing laws and regulations that limit students' ability to chew khat is crucial to reducing the number of users and the further consequences of khat chewing. Thus, to reduce the prevalence of khat use and its negative effects on society, the economy, and health, the Minister of Education must collaborate with the Ministry of Health to integrate life skill training into the curricula for secondary school students. A place for open discussion should also be created to raise awareness of the harmful effects of khat and urge behavioral adjustment. It is also necessary to outlaw the sale of khat to adolescents and young adults, encourage khat users to seek medical attention and advocate for support services for khat users among their peers.

## Data availability statement

The original contributions presented in the study are included in the article/supplementary material, further inquiries can be directed to the corresponding author.

## Ethics statement

The studies involving humans were approved by Haramaya University College of Health and Medical Science Institutional Health Research Ethics Review Committee (IHRERC). The studies were conducted in accordance with the local legislation and institutional requirements. The Helsinki Declaration on Medical Research Ethics guided the conduct of the study (65). Written informed consent for participation in this study was provided by the participants' legal guardians/next of kin. Written informed consent was obtained from the individual(s) for the publication of any potentially identifiable images or data included in this article.

## Author contributions

All authors listed have made a substantial, direct, and intellectual contribution to the work and approved it for publication.
